# Long term follow-up of multiorgan disease in Kleefstra syndrome 2 in an adult – case report

**DOI:** 10.1186/s12883-025-04210-8

**Published:** 2025-05-06

**Authors:** Zhiyong Chen, Jia Liang Kwek, Ru Sin Lim, Yan Rong Yong, Alwin Hwai Liang Loh, Weng Khong Lim, Jing Xian Teo, Karine Su Shan Tay, Peng Soon Ng

**Affiliations:** 1 Department of Neurology, National Neuroscience Institute (Tan Tock Seng Hospital Campus), 11 Jalan Tan Tock Seng, Singapore, 308433 Singapore; 2 Department of Renal Medicine, Singapore General Hospital, Singapore, Singapore; 3 Department of Renal Medicine, Tan Tock Seng Hospital, Singapore, Singapore; 4 Department of Diagnostic Radiology, Changi General Hospital, Singapore, Singapore; 5 Department of Anatomical Pathology, Singapore General Hospital, Singapore, Singapore; 6 SingHealth Duke-NUS Institute of Precision Medicine, Singapore, Singapore

**Keywords:** Neurodevelopmental disorder, Mitochondrial disease, Kleefstra syndrome, *KMT2 C* gene, Focal segmental glomerulosclerosis, Case report

## Abstract

**Objectives:**

The Kleefstra syndrome spectrum (KSS) is a group of neurodevelopmental disorders characterized by intellectual disability, behavioral disorders, growth and neurodevelopmental delay, facial dysmorphism and neurological deficits. Kleefstra syndrome 2 (KLEFS2) is a part of KSS and is due to heterozygous loss-of-function variants in the *KMT2 C* gene. We report the long-term clinical course and multi-organ manifestations of a patient with KLEFS2 caused by a novel heterozygous pathogenic variant in *KMT2 C*.

**Methods:**

A patient with KSS phenotype developed proteinuria with progressive kidney dysfunction secondary to focal segmental glomerular sclerosis. She subsequently developed recurrent episodes that mimicked mitochondrial stroke-like episodes. The phenotype included encephalopathy, stroke-like episodes with focal status epilepticus with impaired consciousness associated with cortical and subcortical T2/FLAIR signal hyperintensities that partially responded to intravenous arginine infusions.

**Results:**

Exome sequencing revealed a heterozygous pathogenic nonsense variant in *KMT2 C* (NM_170606.3) c.3940C > T (p.Gln1314Ter). Nuclear and mitochondrial DNA variants associated with mitochondrial disorders have been excluded.

**Discussion:**

This is a case of KLEFS2 with longitudinal 10 year follow up and its previously unreported multi-organ clinical manifestations including stroke-like episodes and nephrotic disease. Our report further expands the phenotypic spectrum of KLEFS2. Further reports of patients with KLEFS2 with multi-organ involvement should be sought to confirm our findings.

**Supplementary Information:**

The online version contains supplementary material available at 10.1186/s12883-025-04210-8.

## Introduction

The Kleefstra syndrome spectrum (KSS) is a group of rare neurodevelopmental disorders characterized by clinical features which include intellectual disability, behavioral disorders, growth and neurodevelopmental delay, facial dysmorphism and neurological deficits. Genes so far implicated with the KSS phenotype include *EHMT1, KMT2 C, SMARCB1* and *MBD5* [1]. These genes encode histone methyltransferases that are involved in the regulation of chromatin structure which in turn regulate gene expression. Kleefstra syndrome 2 (KLEFS2, OMIM: 617768), is caused by heterozygous loss-of-function variants in the *KMT2 C* gene. We report the long-term clinical course and multi-organ clinical features of a patient with KLEFS2 with a heterozygous nonsense variant in *KMT2 C*.

## Case summary

A Chinese woman first presented at the age of 46 years (2014) with recurrent unilateral headaches associated with transient homonymous hemianopia. These headache episodes were not responsive to migraine prophylaxis trials of tricyclic antidepressants, calcium channel blockers and valproic acid.

**Fig. 1:**
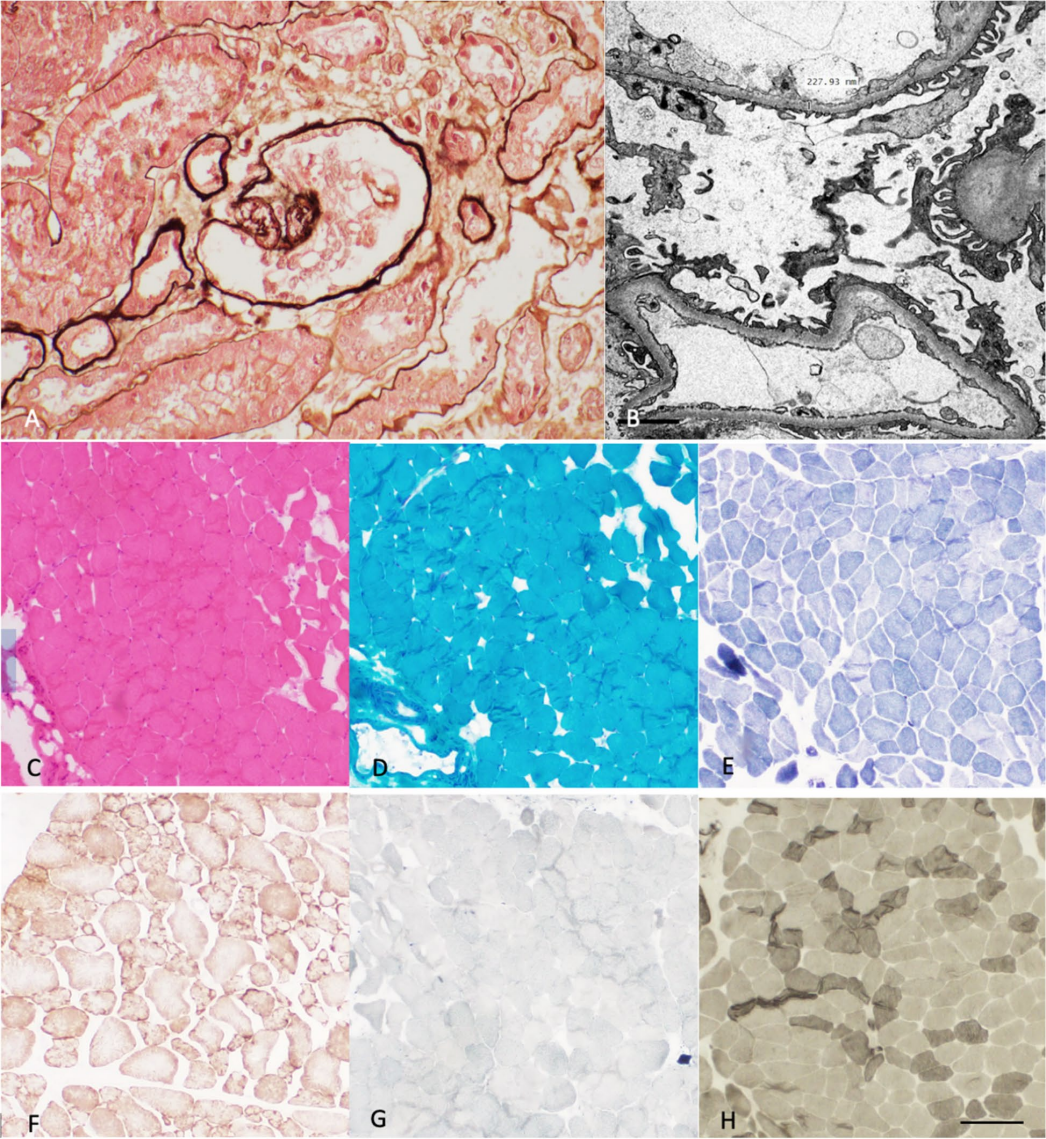
Kidney biopsy (**A**) reveals collapsed glomerular capillary tufts associated with epithelial cell hyperplasia in keeping with focal segmental glomerular sclerosis, (**B**) electron microscopy reveals some degree of mild podocyte foot process effacement. There are no electron dense deposits to indicate immune complex mediated glomerular disease. Biceps brachii muscle biopsy (**C**) There is mild variation in fibre size. **D** This are no ragged red fibres or abnormal inclusions seen. is associated with the predominance of type 1 fibres associated with predominant type 2 fibre atrophy. **E** Myofibrillar architecture is normal. **F** There are no COX negative SDH positive fibers. **G** Sudan Black stains are normal. **H** A mosaic pattern of type I and II fibers is maintained. (**A** Masson-silver stained section, original magnification × 400, **C**: Hematoxylin and eosin (HE) stain, original magnification × 4; **D**: Gömöri-trichrome stain, original magnification × 4; **E**: NADH-TR stain, original magnification × 4; **F**: COX/SDH stain, original magnification × 4; **G**: Sudan black stain, original magnification × 4; **H**: ATPase pH9.4, original magnification × 4; **C**-**H**: scale bar 200 μm)

Two years after initial presentation (2016), she developed hypertension with nephrotic range proteinuria. Kidney biopsy showed focal segmental glomerular sclerosis, ultrastructural examination revealed partial effacement of podocyte processes, normal mitochondrial morphology with no evidence of an underlying inflammatory process (Fig. 1A, B)). Laboratory evaluation for infectious (HIV, hepatitis B, hepatitis C, syphilis), autoimmune etiologies (ANA, anti-ENA, anti-ds-DNA, anti-cardiolipin antibodies, lupus anticoagulant, C3 and C4) and primary amyloidosis were unremarkable. Proteinuria and renal function worsened despite the use of a renin-angiotensin blocking agent. Use of corticosteroids resulted in infections, necessitating its cessation.

**Fig. 2:**
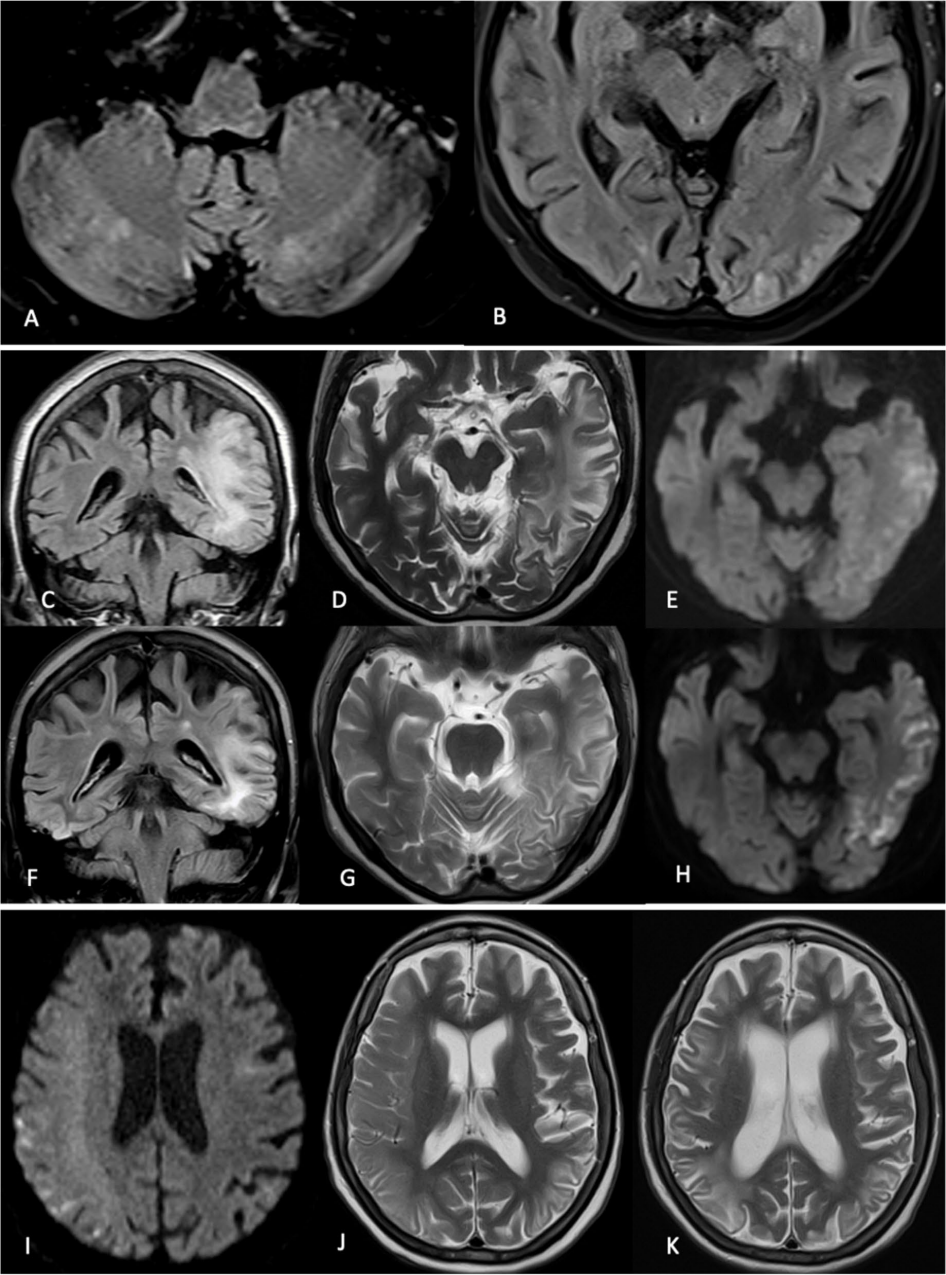
MRI Brain August 2017 FLAIR images showing (**A**) subtle bilateral cerebellar and (**B**) bilateral occipital cortical and subcortical white matter hyperintensities. These findings were thought to represent posterior reversible encephalopathy syndrome (PRES). MRI Brain in March 2019 (**C**, **D**, **E**) shows (**C**) left temporal and left parietal cortical and subcortical white matter FLAIR hyperintensities with vasogenic oedema, (**D**) left temporal and left occipital T2-weighted hyperintensities. **E** corresponding areas of patchy DWI hyperintensity. These new changes in the left cerebral hemisphere resemble stroke-like changes secondary to mitochondrial encephalopathy with stroke-like episodes (MELAS). Corresponding MRI Brain in June 2019 (**F**, **G**, **H**) shows temporal evolution of the changes observed in (**C**, **D**, **E**) with interval reduction of vasogenic oedema, stable DWI signal and interval development of gliotic changes. MRI Brain in March 2021 reveal right fronto-parieto-occipital cortical and subcortical white matter (**I**) DWI hyperintensity and (**J**) T2 hyperintensity with vasogenic oedema. MRI Brain in January 2022 reveal (**K**) interval development of gliotic changes in the right cerebral hemisphere. Note the pre-exisiting gliotic changes in the left cerebral hemisphere secondary to the stroke-like episode in March 2019. (**A**, **B**) Axial FLAIR; (**C**, **F**) Coronal FLAIR; (**D**, **G**, **J**, **K**) Axial T2-weighted; (**E**, **H**) Axial DWI

A year later (2017), she presented with headache, encephalopathy and hypertensive urgency. MRI brain was consistent with the diagnosis of posterior reversible encephalopathy syndrome (PRES) (Fig. 2A, B) and cerebrospinal fluid (CSF) evaluation was normal. Her mental status improved after normalization of her blood pressure.

Neurologic examination at baseline was normal apart from generalized areflexia and hypotonia. Of note she had short stature (4.5 feet (1.37 m)), hypertelorism and mild mid-face hypoplasia. She had mild intellectual disability based on the DSM-5 criteria with deficits in executive function, abstraction, language and short-term memory. She required some support with complex living tasks. She was however able to work as a janitor. Her early developmental history could not be obtained.

The patient’s mother developed dementia at the age of 50 years old. The patient was the eldest of four siblings (all females). Medical histories of her siblings could not be obtained. She was married with no children.

She subsequently had a complicated clinical course over the next 6 years. One month after the previous admission, she presented with headache, encephalopathy accompanied by left gaze deviation and left hemiplegia. Non-contrast MRI brain showed increased T2/FLAIR signal along bilateral occipital cortex (images unavailable). EEG showed electrographic seizures in the right hemisphere and serum lactate of 2.77 mmol/L (normal range: 0.50–2.20 mmol/L).

The clinical presentation resembled a restricted form of MCARNE (Mitochondrial cerebellar ataxia, renal failure, neuropathy and encephalopathy) [2]. She was evaluated for mitochondrial disease: CSF evaluation was unremarkable, CSF lactate (2.71 mmol/L), biceps muscle biopsy (Fig. 1C-H) and transthoracic echocardiography were unremarkable. Nevertheless, she was initiated on oral Coenzyme Q10, phenytoin and levetiracetam. Her mental state gradually improved and she was able to return to her previous occupation.

Two years later (2019), she developed acute onset of receptive aphasia with cognitive decline. MMSE score was 9. MRI brain showed confluent T2/FLAIR hyperintensity in the left parietal, temporal and occipital lobes without contrast enhancement (Fig. 2C-E). EEG revealed continuous slow waves over the left hemisphere. Autoimmune encephalitis panel (Anti-NMDA, LGI1, CASPR2, DPPX, GABAA and MOG antibodies) was negative. Intravenous (IV) arginine was administered. Patient demonstrated clinical improvement within a week with reversion of MMSE to baseline of 20 and partial resolution of initial radiological changes 3 months later (Fig. 2F-H).

Approximately a year later (in 2021), she presented with a sudden onset of left-sided body weakness, encephalopathy and myoclonic jerks. MRI brain showed right fronto-temporo-parietal T2/FLAIR hyperintensity with swelling and DWI hyperintensity with restricted diffusion (Fig. 2I, J). EEG did not show any electrographic seizures. She was initiated on IV arginine with improvement in her mental state and resolution of myoclonic jerks.

Over the next 3 years, she suffered recurrent nosocomial infections and worsening of her renal function necessitating recurrent intensive care admissions and eventual renal replacement therapy with hemodialysis. She also developed recurrent stroke-like episodes with stepwise deterioration of her cognition. MRI brain (2022) showed gliotic changes in both hemispheres (Fig. 2K).

Subsequent whole exome sequencing showed novel heterozygous pathogenic nonsense variant in *KMT2 C* (NM_170606.3) c.3940 C > T (p.Gln1314 Ter). Mitochondrial genome and nuclear encoded mitochondrial genomic analysis from whole blood was unremarkable. It was not possible to obtain tissue for chromosomal microarray studies. She eventually died of nosocomial sepsis at the age of 56.

## Methods

### Patient recruitment and ethical consideration

Written informed consent for publication of case details was obtained. Genetic counselling was performed in accordance with local guidelines. The research was approved by the SingHealth Institutional Review Board (ID 2018/2341).

### Whole exome sequencing

Genomic DNA was isolated from the patient’s whole blood. Samples were prepared using the IDT xGen Exome Research Panel V1.0 (IDT). ENA sample was sheared adaptor ligated PCR amplified and incubated with the exome baits. Captured DNA was eluted and PCR amplified. Final quantified libraries were seeded onto an Illumina flow cell and sequenced using paired-end, 150 cycle chemistry on the illumina NovaSeq, NextSeq or HiSeq. Initial data processing, base calling, alignments and variant calls were generated by various bioinformatics tools using genome assembly GRCh 37. Data was annotated with the Ambry Variant Analyzer tool. All relevant findings underwent manual review by molecular geneticists using IGV and undergo confirmation by Sanger sequencing.

### Mitochondrial genome sequencing

Genomic DNA was isolated from the patient’s whole blood. PCR amplification and Illumina next-generation sequencing (NGS) was used to test for the presence of variants within the mitochondrial genome (includes 13 protein coding genes, 22 transfer RNA genes, and 2 ribosomal RNA genes). Large deletions within the mitochondrial genome were identified by gel electrophoresis and deletion breakpoints were then determined from NGS data.

Muscle and renal biopsies that were performed followed routine histopathology conducted as part of the clinical work-up. Investigations included H&E, Gömöri trichrome, NADH staining, Sudan black and ATPase pH4.3 and ATPase pH9.4. Renal ultrastructural analysis was carried out on longitudinal and transverse ultrathin sections after staining with uranyl acetate and lead citrate, using a transmission electron microscope.

All MRI brain studies were conducted as part of standard clinical care using either 1.5 T or 3.0 T clinical scanners. Pulse sequences performed included T1 weighted imaging, diffusion-weighted imaging (DWI), fast spin-echo T2-weighted imaging or fluid-attenuated inversion recovery (FLAIR), and gradient-recalled echo (GRE).

## Discussion and conclusions

We report the long-term clinical course and extra-neurological features of a patient with Kleefstra syndrome spectrum (KSS) phenotype caused by a heterozygous pathogenic nonsense variant in *KMT2 C* (NM_170606.3) c.3940 C > T; p.Gln1314 Ter. This variant was previously reported in Clinvar without substantiation of disease phenotype. This variant is predicted to lead to nonsense-mediated mRNA decay resulting in haploinsufficiency of the *KMT2 C* gene. The majority of published pathogenic variants in *KMT2 C* are loss-of-function variants in the form of nonsense mutations, frameshift mutations and deletions. We have additionally excluded nuclear and mitochondrial DNA variants associated with mitochondrial disorders.

Genes that are associated with the KSS phenotype include *EHMT1, KMT2 C, SMARCB1* and *MBD5* [1]. Kleefstra syndrome 1 (KLEFS1, OMIM: 610253) is caused by heterozygous microdeletions in the *EHMT1* gene or 9q34.3 deletions encompassing the *EHMT1* gene. Extra-neurological clinical features including renal, urological, cardiac and genital manifestations have been reported to occur in KLEFS1 [3]. There are few published reports of patients with KLEFS2, OMIM: 617768, a summary of clinical features is tabulated by Whitford et al. [4]. All of which reported paediatric cases or young adults with mainly neurodevelopmental manifestations and relatively short follow-up durations [4–12].

The *KMT2 C* gene encodes the histone-lysine N-methyltransferase 2 C (KMT2 C) protein. KMT2 C forms the enzymatically active Set1 domain of the protein complex termed complex proteins associated with Set1 (COMPASS). This complex catalyses the methylation of histone H3 at the lysine-4 position (H3 K4 me1). The methylation status of histones regulates chromatin which in turn influences gene transcription [13, 14].

Both *EHMT1* and *KMT2 C* share similar biological functions and interact with one another. *EHMT1* expression also exerts an inhibitory effect on *KMT2 C* expression [1].

In neuronal tissue, *KMT2 C*, *EHMT1, SMARCB1* and *MBD5* deficiency results in the differential expression of genes associated with the regulation of neuronal excitability and synaptic function. This differential gene expression had been shown in cell models to result in increased neuronal cell excitability as well as the development of hyperexcitable neuronal networks [15].

*KMT2 C* is widely expressed in human tissues and fetal brain tissue[16]. It has been demonstrated through ChIPseq that *EHMT1* and *KMT2 C* analogues in *Drosophila* directly targets the expression of the genes *pyruvate carboxylase* (*PCB*), and *mitochondrial transcription termination factor* (*mTTF*). Both *PCB* and *mTTF* are genes involved in mitochondrial metabolism [5, 17]. Through the use of KMT2 C knock-out mouse models Erbb2/Neu, Myc or PIK3 CA driven tumorigenesis, Simigdala et al. observed that loss of KMT2 C results in mitochondrial dysfunction [18]. Additionally Schon et al. identified a subject with a pathogenic loss-of-function variant of *KMT2 C* from a cohort of patients with suspected mitochondrial disorders [19]. It can thus be extrapolated that KMT2 C deficiency may result in a disorder with multiple organ involvement and simulate a mitochondrial disorder.

There are few reports of adult patients with KSS (summary of cases listed in supplemental table). Verhoeven et al. reported a 43 year-old patient with KLEFS1 with neurodevelopmental disorder, who developed progressive cognitive impairment, new onset epilepsy and cardiac impairment, simulating a mitochondrial syndrome [20]. Given additionally the observation that Klefs1-*EHMT1*-related disease results in a multisystemic disorder which includes cardiac and renal involvement, it can be extrapolated that KMT2 C deficiency will result in a disorder with multiple organ involvement as well.

Analysis of the GnomAD database reveals the presence of several subjects harboring heterozygous loss-of-function variants in *KMT2 C* within the control population [21]. These findings suggest that certain heterozygous loss-of-function variants in *KMT2 C* may result in reduced disease penetrance or variable phenotypic expressivity. This in turn results in a milder disease phenotype, leading to the underdiagnosis of KLEFS2. This is reflected in our patient, who was mildly affected at a younger age and only developed severe disease sequelae later in life.

Our patient received a delayed genetic diagnosis as her neurodevelopmental disorder was not initially recognised. Moreover, the patient’s clinical presentation resembled MCARNE, a mitochondrial disease not previously associated with KLEFS2. The abovementioned two diagnostic gaps may be bridged through firstly deep phenotyping in collaboration with a multidisciplinary team involving a clinical geneticist and organ-specific experts. Secondly, the utilisation of exome or genome sequencing in the context of a dysmorphic patient or a patient with multi-organ manifestations. Lastly, recognizing a genetic cause should be considered despite absence of family history and clinical onset in later adulthood.

This study is limited by our inability to identify additional patients with KLEFS2 with multiple organ involvement. We were also unable to obtain additional tissue for functional studies and perform chromosomal microarray studies. We have however evaluated this patient extensively to exclude additional disease aetiologies. The patient’s initial neurodevelopmental manifestations can also be sufficiently explained by her loss-of-function *KMT2 C* variant.

Our report further expands the phenotypic spectrum associated with KLEFS2.

## Supplementary Information

Supplementary Material 1. Supplemental table: Reported adult patients in literature with Kleefstra syndrome spectrum.

## Data Availability

The datasets generated and/or analysed during the current study are available in the ENA repository, Accession number: PRJEB87498.

## Abbreviations

CSF: Cerebrospinal fluid
KLEFS1: Kleefstra syndrome 1
KLEFS2: Kleefstra syndrome 2
KMT2 C: Histone-lysine N-methyltransferase 2C
KSS: Kleefstra syndrome spectrum
MCARNE: Mitochondrial cerebellar ataxia, renal failure, neuropathy, and encephalopathy
MMSE: Mini mental state examination
PRES: Posterior reversible encephalopathy syndrome
