# Supplementary material for: Long term follow-up of multiorgan disease in Kleefstra syndrome 2 in an adult – case report
Source: BMC Neurol. 2025 May 6;25:199. doi: 10.1186/s12883-025-04210-8 (PMC12057049; doi:10.1186/s12883-025-04210-8)
Supplement: Supplementary file 1 — Supplementary Material 1. Supplemental table: Reported adult patients in literature with Kleefstra syndrome spectrum. [file 12883_2025_4210_MOESM1_ESM.docx]

| Author,  year of publication | Number of  patients | Condition | Age | Clinical features |
| --- | --- | --- | --- | --- |
| Kleefstra 2006 | 1 | KLEFS1 | 36 | Neurodevelopmental  Brachydactyly, seizures |
| Verhoeven 2009 | 2 | KLEFS1 | 53, 59 | Behavioral and neuropsychiatric |
| Verhoeven 2011 | 3 | KLEFS1 | 43, 33, 19 | Neurodevelopmental disorder, patients developed progressive cognitive impairment and  1 patient developed epilepsy, and cardiac arrhythmias  1 patient developed progressive limb rigidity |
| Koemans, 2017 | 2 | KLEFS2 | 29, 31 | Neurodevelopmental, kyphoscoliosis  1 patient had crypto-orchidism |

Supplemental table: Reported adult patients in literature with Kleefstra syndrome spectrum
